# The Long-Term Influence of Body Mass Index on the Success Rate of Mid-Urethral Sling Surgery among Women with Stress Urinary Incontinence or Stress-Predominant Mixed Incontinence: Comparisons between Retropubic and Transobturator Approaches

**DOI:** 10.1371/journal.pone.0113517

**Published:** 2014-11-21

**Authors:** Seong Jin Jeong, Han Sol Lee, Jeong Keun Lee, Jin Woo Jeong, Sang Cheol Lee, Jeong Hyun Kim, Sung Kyu Hong, Seok-Soo Byun, Sang Eun Lee

**Affiliations:** 1 Department of Urology, Seoul National University Bundang Hospital, Seongnam, Korea; 2 Department of Urology, Kangwon National University Hospital, Chuncheon, Korea; Oslo University Hospital, Ullevål, Norway

## Abstract

**Objectives:**

Mid-urethral sling (MUS) surgery for the treatment of urinary incontinence has been widespread since the introduction of tension-free vaginal tape in the mid-1990s. The majority of studies with short-term follow-up <2 years found no differences in the surgical outcomes according to body mass index (BMI). However, considering the chronic influence of obesity on pelvic floor musculature, it is cautiously speculated that higher BMI could increase stress on pelvic floor and sub-urethral tape, possibly decreasing the long-term success rate in the obese population. We aimed to compare the long-term effects of BMI on the outcomes of MUS between women with retropubic and transobturator approaches.

**Methods:**

We performed a retrospective analysis on 243 consecutive women who received MUS and were followed up for ≥36 months. The influence of BMI on the success rates was separately estimated and the factors for treatment failure were examined using logistic regression in either approach.

**Results:**

The mean follow-up was 58.4 months, and 30.5% were normal weight, 51.0% overweight, and 18.5% obese. Patients received either the retropubic (30.5%) or transobturator (69.5%) approach. The success rates (%) under the transobturator approach differed according to the BMI groups (94.3, 88.6, and 78.6, respectively; P = 0.037) while those under the retropubic approach were not different according to the BMI groups. However, in multivariate models, only the presence of preoperative mixed urinary incontinence (MUI) was proven to be the risk factor for treatment failure in the transobturator approach (OR 6.39, P = 0.003). The percent of subjects with MUI was higher in obese women than in non-obese women with the transobturator approach.

**Conclusions:**

BMI was not independently associated with failures in either approach. Higher success rates in women with lower BMI in the transobturator approach were attributed to the lower percent of preoperative MUI in those with lower BMI.

## Introduction

Obesity is a growing health burden all over the world. It influences more than 25% of adults in the United States and in European countries [1], [2]. Asians tend to exhibit a greater percentage of upper body subcutaneous fat than do Caucasians, although they tend to have lower body mass index (BMI) values than Caucasians [3]. Obesity may affect the pelvic floor musculature due to the increased intra-abdominal pressure or neurophysiological mechanism [4], [5]. Furthermore, the association of overweight and obesity with urinary incontinence has been well described in women [6].

Mid-urethral sling surgery has been widespread since the introduction of tension-free vaginal tape in the mid-1990s [7]. Except for particular populations such as those with intrinsic sphincter deficiency, the retropubic and transobturator approaches currently demonstrate equivalent efficacy across a number of randomized controlled trials [8]. The majority of studies with short-term follow-up <2 years found no significant differences in the surgical outcomes according to BMI [4], [9]–[13]. However, considering the chronic influence of obesity on the pelvic floor musculature that leads to urinary incontinence [5], it is cautiously speculated that higher BMI could increase stress on the pelvic floor and sub-urethral tape itself during physical activities after surgery, possibly contributing to the position of the tape into a looser situation in relation to the urethra and thus decrease the long-term success rate in the obese population. Therefore, a longer follow-up may be required to reinforce the findings with regard to the association of obesity with outcomes of mid-urethral sling surgery. Until now, only two studies have followed up their subjects for ≥5 years following surgery to evaluate the influence of obesity [14], [15]. These studies demonstrated contradictory results in women who underwent surgery via the retropubic approach.

We hypothesized that the influence of obesity on the long-term success would differ between women who underwent either the retropubic and transobturator approach, because the retropubic approach would provide a more vertical and more circumferential position of the tape than the transobturator approach against chronic stress on the pelvic floor in obese women. Therefore, the retropubic approach would support the urethra better over the long term for obese patients than the transobturator approach. The purpose of the present study was to compare the effects of BMI on the long-term outcomes of mid-urethral sling surgery between subjects with retropubic and transobturator approaches in Korean women who were followed-up for ≥36 months after surgery.

## Materials and Methods

### Study population

From October 2003 to December 2008, 402 consecutive women underwent mid-urethral sling surgery other than tension-adjustable sling for stress urinary incontinence or stress-predominant mixed urinary incontinence (MUI), defined clinically based on the report from the International Continence Society [16], at our institution. Of these, 243 who were followed-up for at least 36 months were retrospectively enrolled into the analyses. All clinical data including demographic characteristics, severity of incontinence [17], surgical procedures, complications [18] and follow-up tests were collected from a computerized database. The Institutional Review Board of Seoul National University Bundang Hospital (Seongnam, Republic of Korea) approved the study protocol based on the Declaration of Helsinki. The approval number is B-1301/188-109 and we were given exemption from getting informed consents by the Institutional Review Board. All personal identifiers were removed from the data and the data were anonymously analyzed.

According to the specified protocol for women with urinary incontinence at our institution, history taking, physical exam, validated Incontinence Quality of Life questionnaire (I-QOL) [19], 3-day frequency-volume chart including the Urinary Sensation Scale at every voiding, urinalysis, urine flow study (DABA, Endo Tech, Seongnam, Korea) with measurement of post-void residual (PVR; BladderScan BVI-3000, Diagnostic Ultrasound, USA) were performed initially. The Urinary Sensation Scale is a five-point scoring system describing the degree of urgency felt by the patient ranging from 1 (no urgency) to 5 (urgency incontinence) [20]. MUI was clinically defined as the condition of involuntary leakage associated with urgency and also with exertion, effort, sneezing or coughing based on the report from the International Continence Society [16] using patient symptoms and one or more Urinary Sensation Scale rating 5 on a 3-day frequency-volume chart. Stress-predominant MUI was determined based on the subjective judgment of patient and physician when stress urinary incontinence was more severe and bothersome than urgency incontinence in the real-life of patient. Then, stress test, 1 hour-pad test, and urodynamic study were performed in all patients before mid-urethral sling surgery. The stress test was conducted in the 45° sitting or standing position at the amount of half functional bladder capacity. Usually 3–4 times of straining and cough were performed, but if there was no evidence of urine leakage, then we instructed the patient to jump in place 3–4 times to identify urine leakage. These tests were conducted in the urology laboratory by a specially trained nurse. A multichannel urodynamic study (UD-2000, Medical Measures System, The Netherlands), including a pressure-flow study, were carried out according to the guidelines of the International Continence Society [21]. Patients were instructed to discontinue any drugs possibly affecting micturition function at least 1 week prior to undergoing a urodynamic study. The idiopathic detrusor overactivity was regarded as positive if spontaneous or provoked involuntary detrusor contraction was observed in the filling cystometry [21]. Valsalva leak point pressure was measured in the sitting or standing position at the amount of half maximal cystometric capacity.

Mid-urethral sling surgery was carried out via either the retropubic or transobturator approach at the discretion of physicians with discussions with patients. Surgeries were carried out by a total of four surgeons in the present study. However, most of the surgeries (93.4%) were carried out by two surgeons (SJJ and SEL). All patients showed urine leakage during Valsalva maneuver on urodynamic study. Surgery was performed based on the standard technique described by Ulmsten et al [22] or Delorme et al [23], with some modifications. TVT (Gynecare, Ethicon, Somerville, USA) or SPARC sling (American Medical System, Minnealpolis, USA) was used in the retropubic procedure, and TVT-O (Gynecare, Ethicon, Somerville, USA), MONARC (American Medical System, Minnealpolis, USA), or SMESH (WooRhi Medical, Namyangju, Korea) was used in the transobturator procedure.

Follow-up consisted of physical exam, completion of validated symptom questionnaire, 3-day frequency-volume chart (if necessary), stress test, and urine flow with measurement of PVR. Patients were followed up at 1, 3, 9, 15 months after surgery and yearly thereafter. Intraoperative and postoperative complications were examined.

### Variable definitions

Treatment success was defined as ‘cured’ (absence of subjective complaint of leakage and absence of objective leakage on the stress test) or ‘improved’ (rare leakage subjectively, but satisfaction regardless of the stress test) through the patient interview and the stress test by a physician or medically qualified research assistant. All other outcomes and use of any treatment for postoperative incontinence were considered as failures. The type of persistent or recurrent incontinence was not concerned in the judgment of treatment outcomes because urinary incontinence itself may be more relevant to patients regardless of the type of incontinence such as stress or urgency. Subjective satisfaction about the treatment was surveyed as follows: ‘very satisfied’, ‘satisfied’ and ‘unchanged/dissatisfied’.

Women were categorized according to the baseline BMI. Based on the recommendation by WHO expert consultation [24], BMI cut-off points for public health action for the Asian population were adopted in the present study as follows: <18.5 kg/m^2^ (underweight), 18.5–23.0 kg/m^2^ (normal weight), 23.1–27.4 kg/m^2^ (overweight), and ≥27.5 kg/m^2^ (obese), respectively.

### Statistical analyses

Comparisons among BMI groups were analyzed by a one-way analysis of variance with Scheffe's method for multiple comparisons or linear by linear association depending on the types of variables. The influence of BMI on the success rates and patient satisfactions was separately estimated between women with the retropubic approach and those with the transobturator approach. Finally, logistic regression analyses were performed to identify the factors related to treatment failure in women with either approach. The significance level of two-tailed *P*-value <0.05 in the univariate analysis was a screening criterion for entrance into the multivariate logistic regression to identify independent risk factors. The SPSS software package version 19.0 (IBM Corp, Somers, NY, USA) was used, and a two-tailed P value <0.05 was determined to indicate statistical significance.

## Results

Of the total of 243 women, 74 (30.5%) were normal weight, 124 (51.0%) overweight, and 45 (18.5%) obese according to the BMI assignment. There were no women in underweight category. Seventy-four (30.5%) women received surgery via the retropubic approach and 169 (69.5%) did via the transobturator approach (Table 1). Most of surgeries were performed under spinal anesthesia. However, general or local anesthesia was used in some cases according to co-morbidity of patient or to preference of patient. Spinal (regional) anesthesia was used in 84.6% women, general anesthesia (including monitored anesthetic care) in 12.8%, and local anesthesia in 2.6%. Types of anesthesia were not different according to the type of surgery (approach) or severity of BMI.

**Table 1:** Demographic and urodynamic characteristics according to the body mass index (kg/m^2^) groups.

Characteristics	18.5–23.0	23.1–27.4	≥27.5	P value
No. patients	74	124	45	
Age, years	53.2±1.2^a^	57.1±0.8^b^	59.7±1.6^b^	0.002
Body mass index, Kg/m^2^	21.5±0.1^a^	24.9±0.1^b^	29.3±0.3^c^	<0.001
Duration of symptom, months	69.3±7.9	57.7±5.4	70.9±8.5	0.31
No. vaginal delivery	2.5±0.2^a^	2.8±0.1^a,b^	3.2±0.2^b^	0.03
Previous hysterectomy	11 (14.9%)	24 (19.4%)	8 (17.8%)	0.60
Previous anti-incontinence surgery	4 (5.4%)	7 (5.6%)	3 (6.7%)	0.93
Co-morbid disease^d^	18 (24.3%)	53 (42.7%)	24 (53.3%)	0.001
Menopause without hormone replacement	38 (51.4%)	84 (67.7%)	30 (66.7%)	0.02
Severity of incontinence^e^				0.29
Grade I	50 (67.6%)	79 (63.7%)	27 (60.0%)	
Grade II	23 (31.1%)	42 (33.9%)	16 (35.6%)	
Grade III	1 (1.3%)	3 (2.4%)	2 (4.4%)	
Mixed urinary incontinence	7 (9.5%)	18 (14.5%)	14 (31.1%)	0.003
No. daytime frequency/day	7.3±1.1	8.3±0.8	9.8±1.2	0.29
No. nocturia/day	0.7±0.2	1.2±0.2	1.7±0.5	0.21
Functional bladder capacity, ml	372.7±18.5	365.9±17.9	354.0±17.6	0.58
1-hour pad test, gm	25.1±2.8^a^	28.2±2.8^a,b^	43.6±9.9^b^	0.035
Maximum flow rate, ml/s	26.5±1.3	25.4±0.9	25.1±1.7	0.70
Post-void residual, ml	30.2±4.2	26.5±6.2	29.0±4.7	0.89
Maximal cystometric capacity, ml	386.1±9.7	377.2±7.4	380.2±5.6	0.79
Involuntary detrusor contraction	9 (12.2%)	12 (9.7%)	8 (17.8%)	0.49
Valsalva leak point pressure, cmH_2_O	88.7±2.9	89.6±2.3	94.8±3.0	0.37
Concomitant prolapse repair	3 (4.1%)	5 (4.0%)	1 (2.21%)	0.83
Approach route				0.33
Retropubic	21 (28.4%)	36 (29.0%)	17 (37.8%)	
Transobturator	53 (71.6%)	88 (71.0%)	28 (62.2%)	

Data are expressed as the mean ± standard error or as the number (%).

Comparisons among BMI groups are analyzed by a one-way analysis of variance with Scheffe's method for multiple comparisons or linear by linear association depending on the types of variables.

^a,b,^ and ^c^The same letters indicate non-significant difference.

^d^Co-morbid diseases included hypertension, diabetes, hepatic disease, respiratory disease, and cardiovascular disease that were controlled by medication.

^e^Based on the Stamey classification; Grade I: women who lose urine only with coughing, sneezing, or lifting heavy objects, Grade II: women who lose urine with minimal activity such as walking or arising from the sitting position, Grade III: women who are totally incontinent in the upright position and who cannot hold urine in their bladders.

Intraoperative and postoperative complications are summarized in Table 2. The incidence of each complication was not significantly different between the BMI groups (P>0.05, linear by linear association). Bladder injury during needle passage or significant bleeding requiring transfusion occurred only in women with the retropubic approach. Urinary retention was noted in 5.4, 4.0, and 4.4%, respectively in each BMI group. Of these, one woman with BMI of 18.5–23.0 kg/m^2^ and two subjects with BMI of 23.1–27.4 kg/m^2^ eventually required the procedure for the release of tape tension. During the follow-up, 10.7% of women experienced urinary hesitancy, slow stream or straining, and 4.5% of subjects exhibited de novo urgency. The incidences of these voiding and storage disorders did not differ significantly between the BMI groups (Table 2).

**Table 2:** Intraoperative and postoperative complications according to the body mass index (kg/m^2^) groups.

Complication^a^	18.5–23.0	23.1–27.4	≥27.5	P value
Intraoperative				
Bladder injury	1 (1.4%)	2 (1.6%)	0	0.59
Transfusion	2 (2.7%)	0	0	0.07
Postoperative				
Wound infection	2 (2.7%)	0	1 (2.2%)	0.59
Urinary retention^b^	4 (5.4%)	5 (4.0%)	2 (4.4%)	0.76
Pain on operative site	2 (2.7%)	3 (2.4%)	0	0.36
Hesitancy, slow stream, or straining	12 (16.2%)	9 (7.3%)	5 (11.1%)	0.24
De novo urgency	3 (4.1%)	6 (4.8%)	2 (4.4%)	0.89
Mesh erosion	0	1 (0.8%)	1 (2.2%)	0.20

^a^One or more complications were experienced by 53 women, resulting in an aggregate of 63 recorded complications.

^b^Urinary retention was determined when the patient failed to void after surgery or the volume of PVR was over 300ml after surgery. In these cases, clean intermittent self-catheterization was performed temporarily; however, three patients eventually required the procedure for the release of tape tension.

Comparisons among BMI groups are analyzed by the linear by linear association analysis.

The mean duration of follow-up was 58.4 months (range 36 to 101). The success rate including ‘cured’ (72.0%) and ‘improved’ (16.9%) was 88.9% in all patients, showing no difference among the BMI groups (Table 3, P>0.05, linear by linear association). The subjective satisfaction showed no differences among the groups. When dividing women by the route of approach, the success rates in the transobturator approach became worse with increasing BMI (P = 0.037, linear by linear association), while those in the retropubic approach were not different according to the BMI groups (Table 4, P = 0.06, linear by linear association). The satisfaction rates were not different among the BMI groups in either approach.

**Table 3:** Comparisons of the clinical outcomes and subjective satisfaction according to the body mass index (kg/m^2^) groups in all participants.

	18.5–23.0	23.1–27.4	≥27.5	P value
Clinical outcome				0.51
Success	66 (89.2%)	112 (90.3%)	38 (84.4%)	
Cured	57 (77.0%)	89 (71.8%)	29 (64.4%)	
Improved	9 (12.2%)	23 (18.5%)	9 (20.0%)	
Failure	8 (10.8%)	12 (9.7%)	7 (15.6%)	
Subjective satisfaction				0.93
Very satisfied	42 (56.8%)	63 (50.8%)	26 (57.8%)	
Satisfied	19 (25.7%)	47 (37.9%)	10 (22.2%)	
Unchanged/dissatisfied	13 (17.5%)	14 (11.3%)	9 (20.0%)	
Follow-up, months	60.6 (36–101)	58.4 (36–101)	54.9 (36–95)	0.16

Comparisons among BMI groups are analyzed by the linear by linear association analysis.

**Table 4:** Comparisons of the clinical outcomes and subjective satisfaction according to the body mass index (kg/m^2^) groups in patients with either the retropubic or transobturator approach.

	18.5–23.0	23.1–27.4	≥27.5	P value
Retropubic approach, n	21	36	17	
Clinical outcome				0.06
Success	16 (76.2%)	34 (94.4%)	16 (94.1%)	
Cured	15 (71.4%)	27 (75.0%)	12 (70.6%)	
Improved	1 (4.8%)	7 (19.4%)	4 (23.5%)	
Failure	5 (23.8%)	2 (5.6%)	1 (5.9%)	
Subjective satisfaction				0.12
Very satisfied	8 (38.1%)	20 (55.6%)	11 (64.7%)	
Satisfied	7 (33.3%)	12 (33.3%)	3 (17.6%)	
Unchanged/dissatisfied	6 (28.6%)	4 (11.1%)	3 (17.6%)	
Transobturator approach, n	53	88	28	
Clinical outcome				0.037
Success	50 (94.3%)	78 (88.6%)	22 (78.6%)	
Cured	42 (79.2%)	62 (70.5%)	17 (60.7%)	
Improved	8 (15.1%)	16 (18.1%)	5 (17.9%)	
Failure	3 (5.7%)	10 (11.4%)	6 (21.4%)	
Subjective satisfaction				0.22
Very satisfied	34 (64.2%)	43 (48.9%)	15 (53.6%)	
Satisfied	12 (22.6%)	35 (39.8%)	7 (25.0%)	
Unchanged/dissatisfied	7 (13.2%)	10 (11.3%)	6 (21.4%)	

Comparisons among BMI groups are analyzed by the linear by linear association analysis.

In the multivariate logistic regression models, only the presence of preoperative MUI (clinically-defined) was identified as the risk factor for treatment failure in all patients (Table 5). In women with the retropubic approach, maximal cystometric capacity was identified as the only risk factor although an odds ratio was only 0.99. In contrast, only the presence of preoperative MUI (clinically-defined) was proven to be the risk factor for treatment failure among women with the transobturator approach (Table 5). BMI was not independently associated with failure in either approach. The percent of subjects with MUI was significantly different according to the BMI groups only in women with the transobturator approach (Table 6) while the presence of MUI was not comparable between retropubic and transobturator approaches (25.7% vs 11.8%, P = 0.012).

**Table 5:** Multivariate logistic regression analyses of risk factors for treatment failure in all patients and in those with either the retropubic or transobturator approach.

	OR (95% CI)	P value
All patients		
Age, year	1.03 (0.98–1.07)	0.28
Mixed urinary incontinence	2.92 (1.13–7.58)	0.028
Maximal cystometric capacity, ml	1.00 (0.99–1.00)	0.12
Involuntary detrusor contraction	1.75 (0.59–5.19)	0.31
Body mass index^a^, Kg/m^2^		0.78
18.5–23.0	Reference	
23.1–27.4	0.70 (0.25–1.91)	
≥27.5	0.81 (0.24–2.78)	
Patients with retropubic approach		
Maximal cystometric capacity, ml	0.99 (0.98–0.99)	0.016
Involuntary detrusor contraction	4.90 (0.62–38.49)	0.13
Body mass index^a^, Kg/m^2^		0.15
18.5–23.0	Reference	
23.1–27.4	0.89 (0.34–2.91)	
≥27.5	1.92 (0.64–6.48)	
Patients with transobturator approach		
Age, year	1.04 (0.97–1.12)	0.23
No. of vaginal delivery	1.13 (0.74–1.73)	0.57
Mixed urinary incontinence	6.39 (1.88–21.68)	0.003
Body mass index^a^, Kg/m^2^		0.50
18.5–23.0	Reference	
23.1–27.4	1.98 (0.50–7.91)	
≥27.5	2.52 (0.51–12.51)	

The significance level of two-tailed P value <0.05 in the univariate analysis was a screening criterion for entrance into the multivariate logistic regression analysis.

^a^Body mass index was entered into the multivariate analysis regardless of significance in the univariate model because it was main objective variable in the present study.

**Table 6:** Comparisons of the percent of subjects with mixed urinary incontinence according to the body mass index (kg/m^2^) groups in patients with either approach.

	18.5–23.0	23.1–27.4	≥27.5	P value
Retropubic approach, n	21	36	17	
mixed urinary incontinence	3 (14.3%)	11 (30.6%)	5 (29.4%)	0.38
Transobturator approach, n	53	88	28	
mixed urinary incontinence	4 (7.5%)	7 (8.0%)	9 (32.1%)	0.01

Comparisons among BMI groups are analyzed by the linear by linear association analysis.

## Discussion

We investigated whether the influence of obesity on the long-term success of mid-urethral sling surgery differed between women with retropubic and transobturator approaches, as it may be hypothesized that the retropubic approach supports the urethra better over the long term for obese patients than the transobturator approach, because the retropubic approach would provide a more vertical and more circumferential position of the tape than the transobturator approach against chronic stress on the pelvic floor in obese women. Over the mean follow-up period of 58 months, we found that BMI was not independently associated with failures in either approach. Although BMI had an influence on the long-term success under the transobturator approach in the univariate analysis, higher success rates in women with lower BMI in this approach was actually attributed to the lower percent of preoperative MUI in those with lower BMI.

To date, only two European studies have dealt with women who were followed-up for ≥5 years after mid-urethral sling surgery in the evaluation of the influence of obesity [14], [15]. However, both studies dealt with women who underwent only the retropubic approach and demonstrated contradictory results. Among 93 women who were followed up for 7.6 years, Mohamad et al indicated that BMI was not an independent predictor of objective cure rate [14]. Another study conducted by Hellberg et al compared the outcomes between 61 very obese subjects and 291 individuals with normal weight during the follow-up of 5.7 years [15]. The overall cure rate in women with normal weight was 81.2%, which was higher compared to the very obese women (52.1%). This finding is similar to our result that was taken from the univariate analysis in women with the transobturator approach. However, the finding that poor odds ratio for cure was still shown in the very obese women after adjusting for other possible predictors [15] is inconsistent with our result from the multivariate analysis.

Contradictory results among different studies may be attributed to different lengths of follow-up, various clinical definitions of success or failure, or variations in the type of mesh placement around the urethra. As for the variations in the type of mesh placement, no studies have dealt with women with the transobturator approach for a long-term follow-up period of >2 years. Therefore, we compared the long-term influence of BMI on the success rate between women with the retropubic approach and those with the transobturator approach. During the mean follow-up of 58.4 months, we found that while the success rates in women with the retropubic approach might not be different according to the BMI groups, those in women with the transobturator approach differed according to the BMI groups before adjusting for other possible predictors, showing more unfavorable outcomes in obese women. However, multivariate analyses did not prove that BMI was an independent risk factor in either approach route. Instead, only the presence of clinically-defined preoperative MUI was proven to be an independent risk factor for women with the transobturator approach (Table 5). In previous studies, the presence of MUI has also been identified as an independent risk factor for failure of surgery with the retropubic approach [14], [25] or with the transobturator approach [26], [27]. In the present study, the percent of MUI in subjects with BMI of 18.5–23.0 kg/m^2^ was 7.5%, whereas it was 32.1% in those with higher BMI, among women with the transobturator approach (Table 6). Therefore, we believe that a lower success rate in obese women with the transobturator approach in the present study was ascribed to a higher percent of preoperative MUI in this population compared with in non-obese women. In the subjects with the retropubic approach, however, due to the relatively small patient number, it seemed to be uncertain whether the confounding effect of the presence of MUI also had an influence on the success rate, although the percent of subjects with MUI was higher than in women with the transobturator approach. The success rate in women with BMI of 18.5–23.0 kg/m^2^ and the retropubic approach was somewhat modest at 76.2%; this finding may be attributed to the lower percent of the ‘improved’ cases in this group (Table 4). As significant bleeding requiring transfusion occurred in two women and postoperative voiding disorders had a stronger tendency to develop in women with BMI of 18.5–23.0 kg/m^2^ and the retropubic approach, these complications might decrease the ‘improved’ rate. BMI was not statistically associated with the risk for intraoperative and postoperative complications in the present study, though the sample size may not be powered to detect a difference in complications.

In general, the distributions of BMI-based overweight and obesity are different between Asian and Western populations. The mean BMI of women in the present study was 24.6 kg/m^2^ and only 4.5% (11/243) of the subjects had BMI >30 kg/m^2^, which is considered as the cut-off for obesity in the Western population. We used BMI categories of overweight (23.1–27.4 kg/m^2^) and obesity (≥27.5 kg/m^2^) for the Asian population as presented by WHO expert consultation in 2004 [24]. It has been reported that Asians tend to exhibit a greater percentage of upper body subcutaneous fat than do Caucasians, although they tend to have lower BMI values than Caucasians [3]. Therefore, obesity-related health risks may increase with relatively lower BMI values in Asians. We believe that BMI categories used in the present study reflect the influence of obesity on the outcomes of surgery more suitably for Asians. Until now, a few studies involving the Asian population have used these BMI categories [9], [10], [12], however, the follow-up duration was only <2 years. Therefore, the present study had the longest duration of follow-up among the studies with BMI categories for the Asian population.

The present study had the following limitations. First, BMI may overestimate body fat in persons who are very muscular, and it may underestimate body fat in persons who have reduced muscle mass such as the elderly [6]. Whether other measures for body fat, such as waist-to-hip ratio or waist circumference, indicate the same findings is to be elucidated. In addition, for the evaluation of the effects of BMI on the long-term surgical outcomes, it might be appropriate to look at BMI change over time as well as BMI at surgery. Second, we did not include a relevant questionnaire into the analysis. In all the patients, the Korean version of I-QOL [19] was used to evaluate the changes of quality of life after surgery; however, the response rate was not high enough for statistical analysis. Third, two fifths of the subjects who underwent surgery during the study period were not followed up for more than 3 years, and were excluded from the analyses. However, demographics of the excluded patients did not differ statistically compared with those of the study subjects (data not shown), although it remains undetermined whether the excluded patients had a similar nature regarding the influence of obesity on the clinical outcomes. Last, various surgical tapes applied in each group (two different retropubic tapes and three different transobturator tapes) might make the interpretation of results challenging. In addition, there has been some debate about whether the long-term results of mid-urethral sling surgery are similar between the retropubic and the transobturator approach. A recent 5-year results of randomized trial showed no difference in objective and subjective success rates between the retropubic and the transobturator approach [28], whereas another study found that the long-term cure rates for retropubic approach were significantly greater than for transobturator tape in women with intrinsic sphincter deficiency [29]. Although BMI was not independently associated with failures in either approach in our study, differences in the proportion of each approach used in our cohort might influence the results because the relatively small proportion of patients (30.5%) received surgery via retropubic approach.

Our findings were derived from a retrospective investigation in Asian populations within single tertiary center with Asian BMI cut-off points for obesity. Therefore, clinical application should be with caution, and multi-racial and multi-center prospective trials are required to generalize our results.

## Conclusions

Over the mean follow-up period of 58 months, BMI was not independently associated with failures in either approach. Although BMI might have an influence on the long-term success under the transobturator approach, higher success rates in women with lower BMI in this approach was attributed to the lower percent of preoperative MUI in those with lower BMI. Therefore, it should be preoperatively investigated with particular attention in obese women to determine whether subjects have MUI in relation to high BMI, which has been ‘in fact’ proven to be a risk factor for treatment failure.
